# Postbiotics and phage synergy in precision oral microbiome engineering: systems biology strategies targeting *Streptococcus mutans* in dental caries

**DOI:** 10.3389/fmolb.2026.1766853

**Published:** 2026-04-08

**Authors:** Guhanraj Radhamanalan

**Affiliations:** Department of Microbiology, Saveetha Dental college, Chennai, Tamil Nadu, India

**Keywords:** bacteriophages, biofilm, dental caries, multi-omics, oral microbiome, postbiotics, precision therapeutics, *Streptococcus* mutans

## Abstract

Dental caries continues to represent a major global public health concern and arises from complex ecological shifts within oral biofilms. The dominance of *Streptococcus mutans*, in combination with broader microbial imbalance and interactions involving the oral virome, plays a central role in disease progression. Although established preventive measures such as fluoride therapy and mechanical plaque control reduce enamel demineralization and microbial load, they do not comprehensively address dysbiosis, virulence regulation, or host–microbial signaling dynamics. Postbiotics are non-viable microbial products or metabolic derivatives with biological activity, are gaining attention as targeted modulators of the oral ecosystem. These agents include organic acids, exopolysaccharides, bacteriocins, and structural components derived from inactivated probiotic cells. Through diverse mechanisms, postbiotics can reduce acidogenic potential, weaken extracellular matrix integrity within biofilms, disrupt bacterial communication systems, and modulate mucosal immune pathways. Such effects may limit colonization efficiency and pathogenic behavior of *S. mutans* while preserving commensal balance. Emerging strategies propose combining postbiotics with bacteriophage-based approaches, immunomodulatory platforms, and innovative delivery systems such as nanoformulations and bioadhesive matrices to improve site-specific efficacy. Advances in multi-omics technologies, systems biology modeling, and artificial intelligence–driven diagnostics further support the development of personalized interventions tailored to individual microbial signatures. In addition, postbiotic-mediated modulation of viral–bacterial interactions and horizontal gene exchange may contribute to restoring ecological stability and reducing antimicrobial resistance dissemination. This review integrates current knowledge on postbiotic-driven regulation of the oral microbiome and virome and examines their potential role in precision-oriented caries management. Addressing translational challenges, including formulation stability, safety evaluation, regulatory pathways, and comprehensive virome profiling, will be critical for future clinical application.

## Graphical Abstract

**Figure:**
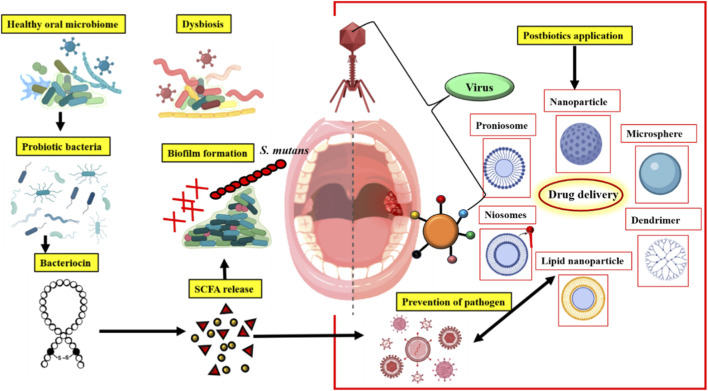

## Introduction

Dental caries is one of the most common chronic diseases worldwide, affecting individuals of all ages and posing a significant public health challenge (Warreth, 2023; Iheozor-Ejiofor et al., 2024). Although largely preventable, the disease burden remains substantial in both industrialized and developing countries, disproportionately affecting children, socioeconomically disadvantaged populations, and communities with limited access to dental care.

Recent research increasingly conceptualizes dental caries as a systems-level disease characterized by complex interactions among biological, behavioral, and environmental factors, rather than as a purely microbial condition (Ribeiro and Paster, 2023). Caries pathogenesis arises from dynamic interactions among the oral microbiome, virome, dietary sugars, salivary factors, and host immune responses, together with genetic determinants that influence enamel integrity and microbial colonization (Rai et al., 2024).

Frequent consumption of fermentable carbohydrates promotes ecological shifts toward acidogenic and acid-tolerant microbial communities, while impaired host defenses further contribute to lesion development. Although traditional preventive strategies such as fluoride application, antimicrobial agents, and mechanical plaque removal have significantly reduced caries prevalence, they do not fully address the underlying ecological dysbiosis within the oral biofilm. Furthermore, antimicrobial resistance, variability in fluoride responsiveness, and patient non-adherence highlight the need for more precise, biologically informed therapeutic interventions (Anil et al., 2023; Ahmed et al., 2025; Moharrami et al., 2024).

Despite growing interest in postbiotics for oral health applications, several critical knowledge gaps remain. The contribution of the oral virome to cariogenic dysbiosis, particularly in relation to horizontal gene transfer, phage-mediated virulence regulation, and microbial resilience, remains insufficiently characterized. Furthermore, mechanistic understanding of how postbiotics influence interconnected metabolic, signaling, and structural pathways within complex biofilms is fragmented, with limited systems-level integration. Translational challenges including delivery optimization, stability, regulatory considerations, and patient-specific variability also remain underexplored. This review addresses these conceptual gaps by synthesizing current evidence through a systems biology perspective, emphasizing microbial–viral interactions, network-based mechanisms, and the potential for precision-guided postbiotic interventions targeting *S. mutans*–associated dysbiosis.

### *Streptococcus* mutans

Among the numerous microorganisms implicated in dental caries, *S. mutans* remains a principal etiological agent due to its distinctive ecological adaptability and genetic versatility. It exhibits strong acidogenic and aciduric properties, enabling survival and proliferation in low-pH environments that inhibit many competing commensal species.

*Streptococcus mutans* produces substantial amounts of extracellular polysaccharides (EPS) through the activity of glucosyltransferases, which facilitate firm adherence to tooth surfaces and enhance the structural integrity and stability of cariogenic biofilms (Zhu et al., 2023; Anil et al., 2023; Banakar et al., 2024). These biofilms are highly resilient, demonstrate reduced permeability to antimicrobial agents, and promote persistent colonization.

**FIGURE 1:**
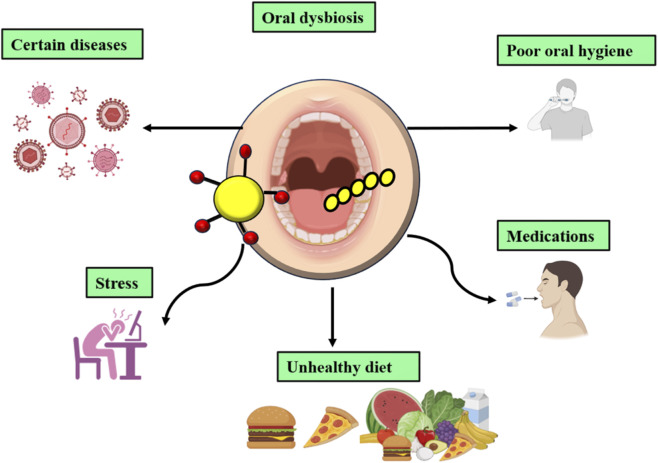
Factors affecting the oral microbiome.

In addition, *S. mutans* exhibits a strong capacity for horizontal gene transfer (HGT), allowing rapid acquisition of adaptive traits, including antimicrobial resistance determinants. Emerging evidence also highlights functional interactions between *S. mutans* and the oral virome, particularly bacteriophages, which may influence strain evolution, virulence gene expression, and stress tolerance (Figure 1). Collectively, these characteristics underpin the ecological dominance of *S. mutans* within cariogenic niches, despite the polymicrobial nature of dentinal caries (Veiga et al., 2023; Iheozor-Ejiofor et al., 2024; Ahmed et al., 2025).

### Postbiotics as precision therapeutics emerges

Advances in microbiome research have stimulated growing interest in postbiotics, defined as non-viable microbial products or metabolic derivatives that confer biological benefits to the host. These include metabolites, bioactive peptides, enzymes, cell wall fragments, exopolysaccharides (EPS), and other structural components generated during bacterial fermentation. Unlike live probiotics, postbiotics do not require survival or colonization within the host environment, offering advantages in terms of safety, stability, controlled dosing, and mechanistic specificity (Fuochi, 2025; Al-Adham et al., 2025; Pattapulavar et al., 2025).

Postbiotics are emerging as precision therapeutics capable of modulating oral biofilm ecology, disrupting pathogenic signaling pathways, and enhancing host defense mechanisms without promoting antimicrobial resistance. Their stability across diverse storage and environmental conditions makes them particularly suitable for incorporation into oral healthcare formulations such as mouthrinses, gels, lozenges, and dentifrices (Dimopoulou et al., 2023; Esmaeilyfard et al., 2024). As research continues to uncover the functional diversity of microbial metabolites, postbiotic-based interventions are increasingly being recognized as promising adjuncts or alternatives to conventional preventive strategies for dental caries.

### The oral microbiome virome network

#### Oral microbiome composition

The oral cavity represents one of the most complex microbial ecosystems in the human body, comprising bacteria, archaea, fungi, and viruses organized within distinct ecological niches, including the tongue, saliva, gingival crevices, and tooth surfaces (Atta et al., 2024; Cervantes-Echeverría et al., 2025). Under healthy conditions, the oral microbiome is dominated by commensal and health-associated species such as *Streptococcus salivarius*, *Streptococcus mitis*, *Veillonella* spp., *Rothia* spp., *Actinomyces* spp., and *Neisseria* spp. These microorganisms contribute to ecological stability by maintaining pH homeostasis, metabolizing lactate, producing nitric oxide, and secreting bacteriocins that suppress opportunistic pathogens. Their metabolic cooperation supports the maintenance of a neutral pH biofilm, thereby protecting enamel and limiting colonization by acidogenic and aciduric species.

Dental caries develops when this ecological balance is disrupted, resulting in microbial dysbiosis. Frequent consumption of fermentable carbohydrates, reduced salivary buffering capacity, and localized acidification selectively favor the proliferation of cariogenic organisms such as *S. mutans*, *Lactobacillus* spp., *Scardovia wiggsiae*, and aciduric *Bifidobacterium* species. Progressive dysbiosis is characterized by reduced microbial diversity, intensified fermentative pathways, and enhanced EPS production. These changes promote the formation of a dense, cohesive, and highly cariogenic biofilm capable of driving enamel demineralization (Howard et al., 2024; de Toledo Guimarães et al., 2023; Zhu et al., 2023).

### Role of the oral virome in microbial ecology

In parallel with bacterial communities, the oral cavity harbors a diverse and dynamic virome dominated by bacteriophages (phages), along with various eukaryotic viruses. Bacteriophages play a critical role in shaping oral microbial ecology by regulating bacterial population density through predation and influencing bacterial evolution via horizontal gene transfer (Tang and Baker, 2025; Spatafora et al., 2024).

During the lytic cycle, phages infect and lyse bacterial cells, thereby altering species abundance and releasing nutrients into the surrounding environment. In contrast, lysogenic phages integrate into bacterial genomes as prophages, where they can modulate host phenotype. Prophage-encoded factors may enhance the ecological fitness of *S. mutans* by increasing acid tolerance, stress resistance, biofilm maturation, and glucosyltransferase activity, thereby strengthening its competitive advantage in acidic environments.

The oral virome also includes eukaryotic viruses such as herpes simplex virus (HSV), human papillomavirus (HPV), Epstein–Barr virus (EBV), and anelloviruses. These viruses can modulate local immune responses, epithelial barrier integrity, and inflammatory signaling pathways. Such interactions indirectly reshape the bacterial ecological landscape, potentially favoring opportunistic and cariogenic organisms, including *S. mutans* and related acidogenic species (Tang et al., 2024; Xu et al., 2025; Esmaeilyfard et al., 2024).

### Interaction between the oral microbiome and virome

The oral cavity constitutes a complex, multi-kingdom ecosystem in which bacteria and viruses interact continuously at genetic, molecular, and ecological levels. Bacteriophages influence bacterial communities not only through predation but also by modulating gene expression, metabolic activity, and stress responses. Integration of prophages into bacterial genomes can activate or repress virulence-associated genes, alter carbohydrate metabolism, and enhance extracellular polymeric substance (EPS) production (Bhagchandani et al., 2024; Meyer et al., 2024).

In *S. mutans*, prophage elements have been associated with increased acid tolerance, improved stress resilience, and enhanced biofilm-forming capacity. Phage-mediated gene exchange represents an important mechanism for disseminating adaptive traits across the oral microbiome. Notably, horizontal gene transfer (HGT) via transduction may facilitate the spread of genes encoding EPS biosynthesis, acid-tolerance islands, stress-response regulators, carbohydrate transport systems, quorum-sensing pathways, bacteriocins, and antimicrobial resistance determinants. As dental caries progresses, both bacterial and viral communities may shift toward a state of co-dysbiosis. This state is characterized by the emergence of more virulent phage populations, a decline in protective commensal phages, reduced microbial diversity, and expansion of pathogenic bacterial species. Such co-dysbiotic interactions reinforce the metabolic efficiency of cariogenic consortia, enhance biofilm resilience, and accelerate enamel demineralization, thereby promoting lesion progression. Understanding this interdependent microbiome–virome axis provides critical insight for designing next-generation strategies aimed at caries prevention and therapeutic modulation (Rajasekaran et al., 2024; Bostanghadiri et al., 2024; Defta et al., 2024; Cervantes-Echeverría et al., 2025).

### Postbiotics as modulators of host pathogen interactions

**FIGURE 2:**
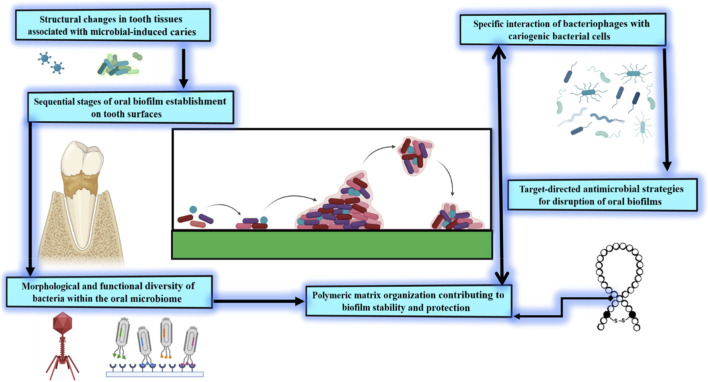
Overview of oral microbial biofilm formation, caries development, and targeted control approaches.

Postbiotics is a non-viable microbial cells, cellular components, or metabolites that confer health benefits have emerged as promising bioactive agents capable of modulating host–pathogen interactions within the oral cavity (Nayak et al., 2025). Unlike live probiotics, postbiotics possess defined mechanisms of action, improved stability, and a reduced risk of translocation-related infection, making them attractive candidates for precision-based caries management (Figure 2).

Accumulating evidence suggests that postbiotics can reshape the ecological landscape of dental biofilms, attenuate virulence pathways in *S. mutans*, strengthen mucosal immune responses, and promote the growth of commensal species. Mechanistically, their effects include disruption of bacterial membrane integrity, interference with quorum-sensing systems, modulation of EPS synthesis, alteration of local pH dynamics, and regulation of host immunological signaling. The following subsections outline key classes of postbiotic molecules relevant to oral health and describe their functional contributions to host–microbe interactions (Fang et al., 2024; Gao et al., 2024; Mazurel et al., 2025; Meyer et al., 2024).

### Postbiotic types of interest in oral health

#### Short chain fatty acids (SCFAs)

Short-chain fatty acids (SCFAs), namely, acetate, propionate, and butyrate, are generated through microbial fermentation of carbohydrates within oral biofilms and function not only as metabolic end-products but also as bioactive signalling molecules. In cariogenic environments, these metabolites influence the physiological fitness of *Streptococcus mutans* by disrupting intracellular pH regulation and interfering with glycolytic enzyme activity, leading to reduced ATP production and impaired carbohydrate metabolism. SCFAs also affect membrane-associated proton transport systems that are essential for acid tolerance, thereby weakening the bacterium’s ability to survive and remain competitive under low-pH conditions. Beyond their microbial effects, SCFAs modulate host responses through activation of G-protein-coupled receptors such as GPR41, GPR43, and GPR109 A expressed on epithelial and immune cells. Engagement of these receptors regulates downstream intracellular pathways, including NF-κB and MAPK signalling, resulting in controlled cytokine production and maintenance of mucosal immune balance. In addition, butyrate exerts epigenetic effects by inhibiting histone deacetylases, which alters chromatin structure and gene transcription patterns associated with epithelial barrier integrity. This promotes the expression of tight junction proteins and supports barrier stability, thereby limiting inflammatory tissue damage. Collectively, SCFAs act as multifunctional mediators that modulate bacterial virulence traits and host inflammatory signalling, highlighting their relevance in strategies aimed at reshaping dysbiotic oral biofilms (Ye et al., 2025; Zhou et al., 2025; Jiang X. et al., 2024).

### Phenyl derived organic acids

Phenyl-derived organic acids produced by lactic acid bacteria, including phenyllactic acid and 4-hydroxyphenyllactic acid, exhibit broad-spectrum antimicrobial activity mediated through cytoplasmic acidification and membrane destabilization. Phenyllactic acid, in particular, has been reported to inhibit bacterial adhesion, interfere with glucosyltransferase activity, and suppress EPS production key virulence determinants of *S. mutans*. Moreover, these metabolites may reduce co-aggregation of cariogenic bacteria while enhancing the ecological competitiveness of health-associated species. Such dual antimicrobial and ecological-modulating effects highlight their potential as targeted postbiotic agents in caries prevention (Heidari et al., 2025; Twetman and Belstrøm, 2025; Salah et al., 2025).

### Antimicrobial peptides and bacteriocins

Ribosomally synthesized antimicrobial peptides, including salivaricin, reuterin-associated peptides, plantaricin, and lactacin, are produced by specific commensal lactic acid bacteria inhabiting mucosal surfaces. Salivaricin is secreted by *S. salivarius*, plantaricin by *Lactobacillus plantarum*, lactacin by *Lactobacillus johnsonii*, and reuterin is generated by *Limosilactobacillus reuteri* during glycerol metabolism. These bioactive compounds exhibit targeted antimicrobial activity primarily against closely related Gram-positive species. In the oral cavity, they demonstrate selective inhibition of cariogenic organisms such as *S. mutans* and *Streptococcus sobrinus*, while generally sparing beneficial commensals including *S. salivarius*. Their antimicrobial mechanisms involve disruption of cytoplasmic membrane integrity through pore formation, dissipation of the proton motive force, interference with nucleic acid and protein synthesis, and modulation of quorum-sensing systems that regulate virulence gene expression. Owing to their narrow spectrum of activity and minimal cytotoxicity toward host tissues, these bacteriocins are increasingly recognized as promising postbiotic candidates for precision-based caries prevention strategies aimed at controlling dysbiosis without broadly disturbing the oral microbiome (Ghamari et al., 2025; Kumar et al., 2024).

The significant postbiotic metabolites include short-chain fatty acids and related bioactive compounds such as ethanoic acid (acetic acid; C_2_H_4_O_2_), propanoic acid (propionic acid; C_3_H_6_O_2_), and butanoic acid (butyric acid; C_4_H_8_O_2_), which contribute to antimicrobial activity and modulation of microbial ecology. Additionally, organic acids with hydroxyl substitutions, including 2-hydroxypropanoic acid (lactic acid; C_3_H_6_O_3_) and 2-hydroxy-3-phenylpropanoic acid (phenyllactic acid; C_9_H_10_O_3_), exhibit bacteriostatic and immunomodulatory properties. Another important bioactive compound is 3-hydroxypropanal (reuterin; C_3_H_6_O_2_), a multifunctional antimicrobial aldehyde known for its broad-spectrum inhibitory effects against oral pathogens.

### Exopolysaccharides (EPS)

Postbiotic EPS derived from *Lactobacillus*, *Limosilactobacillus fermentum*, and *Bifidobacterium* species demonstrate significant immunomodulatory, anti-adhesive, and antibiofilm properties. Depending on their structural composition, these EPS can competitively inhibit pathogen attachment, interfere with biofilm formation, and disrupt the binding of *S. mutans*-derived glucans to tooth surfaces. Certain EPS function as molecular decoys by entrapping pathogens or neutralizing virulence factors. In addition, they may stimulate innate immune responses through activation of pattern-recognition receptors and induction of anti-inflammatory cytokine expression (Al Muhandir et al., 2024; Nizami et al., 2025; Dell'Atti et al., 2025).

In contrast, EPS produced by probiotic bacteria—such as lactobacilli and bifidobacteria—differ in composition and biological function. These polymers are often heteropolysaccharides containing glucose, galactose, rhamnose, or other monosaccharides arranged in branched, less densely packed configurations. Functionally, probiotic EPS generally exhibit reduced adhesive capacity toward enamel and instead interfere with pathogen colonization by modifying surface hydrophobicity, blocking adhesin–receptor interactions, and altering salivary pellicle composition. Some probiotic-derived metabolites additionally downregulate *S. mutans* adhesion-associated genes, including *spaP*, *gbpB*, and *wapA*, thereby impairing co-aggregation and early microcolony formation. Through these structural and functional differences, probiotic EPS tend to exert anti-biofilm and ecological balancing effects, contrasting with the scaffold-forming, virulence-enhancing role of pathogenic EPS. Collectively, distinguishing these structural, compositional, and functional characteristics clarifies how EPS can either promote cariogenic biofilm stability or contribute to its attenuation depending on microbial origin.

## Cell free supernatant (CFS) metabolites

Bacterial cell-free supernatants (CFS) comprise a complex mixture of bioactive molecules, including peptides, low-molecular-weight organic acids, biosurfactants, hydrogen peroxide, reuterin-like compounds, and quorum-sensing inhibitors. CFS-derived components have been shown to suppress the growth of *S. mutans*, inhibit glucosyltransferase activity, reduce acid production, and impair cariogenic biofilm development. Furthermore, CFS may modulate microbial cross-talk by interfering with autoinducer-mediated quorum sensing, thereby limiting interspecies pathogenic cooperation within oral biofilms (Sonmez et al., 2025; Cho et al., 2025).

## Paraprobiotics

Paraprobiotics refer to inactivated microbial cells or cellular components, including peptidoglycans, lipoteichoic acids, and surface-layer proteins. Despite the absence of viability, these bioactive constituents retain immunomodulatory capacity and enhance mucosal defense mechanisms without the potential risks associated with live microorganisms, particularly in high-risk populations. Heat-killed strains of *Lactobacillus* and *Limosilactobacillus fermentum* have demonstrated the ability to reduce *S. mutans* adhesion, attenuate inflammatory signaling, increase salivary immunoglobulin A (IgA) levels, and decrease biofilm acidogenicity. Additionally, they contribute to strengthening epithelial barrier integrity and reducing pathogen translocation (Memè et al., 2024; Cho et al., 2025; Dell'Atti et al., 2025).

## Postbiotics as antimicrobial mechanisms targeting *S. mutans*

### Inhibition of acid production

A key virulence attribute of *S. mutans* is its capacity to ferment dietary carbohydrates into lactic acid, leading to enamel demineralization and enabling survival within low-pH niches. This acidogenic and aciduric phenotype provides a competitive advantage in cariogenic biofilms. Several postbiotic metabolites interfere with this acid-production pathway by inhibiting glycolytic enzymes, reducing intracellular ATP generation, and disrupting transmembrane proton gradients. These metabolites include short-chain fatty acids (SCFAs), organic acids, and bioactive components present in cell-free supernatants. Certain postbiotics derived from lactobacilli have been shown to downregulate acid-associated genes such as *ldh* (lactate dehydrogenase) and *atpD*, which are involved in acid production and acid tolerance mechanisms. By attenuating both acidogenicity and aciduricity, postbiotics create environmental conditions that are less favorable for the persistence and ecological dominance of *S. mutans* within the dental biofilm (Chu and Li, 2025).

### Disruption of the EPS matrix

Cariogenic biofilms are structurally supported by EPS, synthesized primarily through the activity of glucosyltransferases (GtfB, GtfC, and GtfD). These enzymes catalyze the formation of insoluble and soluble glucans that contribute to biofilm cohesion and stability. Various postbiotic molecules including organic acids, structurally distinct EPS, and bioactive peptides present in CFS can disrupt the EPS matrix by inhibiting glucosyltransferase activity, altering glucan architecture, or preventing the polymerization of insoluble glucans (Chen et al., 2024; Salem, 2025). Some postbiotics may directly interact with glucan chains, thereby reducing the mechanical strength and structural integrity of the three-dimensional biofilm matrix. Destabilization of the EPS scaffold compromises the diffusion-barrier properties of the biofilm, enhancing the penetration of salivary antimicrobial factors and increasing the susceptibility of *S. mutans* to mechanical clearance.

### Quorum sensing interference

*Streptococcus mutans*, quorum sensing (QS) regulates genetic competence, bacteriocin production, biofilm maturation, and stress adaptation (Kaur et al., 2026; Palanisamy et al., 2025). The ComCDE and LuxS/AI-2 systems play central roles in intra and interspecies communication. Postbiotics have been reported to interfere with QS pathways through multiple mechanisms, including degradation of signaling molecules, competitive binding to receptors, and downregulation of QS-associated genes. Certain postbiotic peptides structurally resemble natural autoinducers and may function as competitive inhibitors, thereby disrupting coordinated virulence expression. Inhibition of the ComCDE pathway reduces genetic competence and mutacin production, while suppression of LuxS-mediated AI-2 signaling attenuates interspecies communication and impairs biofilm organization (Zhang F. et al., 2025; Banar et al., 2024; Claisse et al., 2025).

### Attenuation of adhesion and biofilm formation

Initial colonization of tooth surfaces by *S. mutans* depends on interactions between bacterial adhesins and salivary pellicle proteins. Postbiotics including biosurfactants, EPS derived from probiotic species, and surface proteins from heat-killed cells—can interfere with this process by modifying surface hydrophobicity, blocking adhesin–receptor interactions, or altering pellicle composition. Certain postbiotic metabolites have been shown to downregulate adhesion-related genes such as spaP, gbpB, and wapA, thereby reducing bacterial attachment to enamel surfaces and limiting coaggregation with other microorganisms. These anti-adhesive effects significantly decrease microcolony formation and delay early biofilm development, ultimately impairing the establishment of a stable cariogenic biofilm (Dallal et al., 2025).

### Bacteriocins and membrane integrity

Bacteriocins represent one of the most potent classes of postbiotics produced by probiotic or commensal bacteria. These ribosomally synthesized peptides exert bactericidal activity primarily through disruption of bacterial membrane integrity. Most bacteriocins form pores in the cytoplasmic membrane of *S. mutans*, resulting in ion leakage, dissipation of membrane potential, and ultimately cell death. Some bacteriocins additionally interfere with cell wall biosynthesis or inhibit essential intracellular processes, including protein translation. Importantly, many bacteriocins exhibit a relatively narrow antimicrobial spectrum, selectively targeting *S. mutans* while sparing beneficial commensals such as *S*. *salivarius*. This specificity enhances their suitability as precision oral therapeutics (Guo L. et al., 2024; Shanmugasundaram et al., 2024).

### Immunomodulatory effects

Postbiotics exert significant immunomodulatory effects within the oral cavity, contributing to mucosal homeostasis and limiting host tissue damage associated with dental caries. Several postbiotic components including SCFAs, bacteriocin-derived peptides, surface-associated molecules, and heat-inactivated microbial cells have been shown to enhance secretory immunoglobulin A (sIgA) production. Elevated sIgA levels strengthen salivary defense mechanisms and reduce early colonization and adhesion of *S. mutans* to enamel surfaces. In addition, postbiotics can attenuate excessive inflammatory signaling during dysbiosis. Experimental evidence indicates that metabolites such as butyrate, phenyl-derived organic acids, and specific paraprobiotic fragments may suppress pro-inflammatory cytokines, including interleukin-1 (IL-1), interleukin-6 (IL-6), and tumor necrosis factor-α (TNF-α), thereby reducing the inflammatory microenvironment that supports cariogenic biofilm progression.

Postbiotics also contribute to epithelial barrier integrity by promoting tight junction assembly, supporting epithelial cell renewal, and modulating Toll-like receptor (TLR) signaling pathways. Collectively, these effects strengthen mucosal defenses, reduce susceptibility to pathogen invasion, and help maintain microbial equilibrium. Thus, postbiotics exert dual protective roles by directly inhibiting *S. mutans* and by modulating the host immune landscape (Luo et al., 2024; Beattie, 2024; Beyrami et al., 2025).

#### Rebalancing oral dysbiosis

Postbiotic-based interventions represent a promising strategy for restoring ecological balance within the oral microbiome, particularly in caries-associated dysbiosis driven by *S. mutans*. Various postbiotic metabolites and cell-derived components have been shown to stimulate the growth and activity of beneficial commensals, including *S*. *salivarius*, *Lactobacillus*, *Rothia*, and *Veillonella* species. These microorganisms play critical roles in maintaining pH homeostasis, generating alkali via the arginine deiminase pathway, and competing with pathogens for adhesion sites. Postbiotics further promote microbial rebalancing through metabolic competition and niche exclusion. Selective inhibition of cariogenic bacteria by bacteriocins, organic acids, and other bioactive molecules allows commensals to recolonize key ecological niches on tooth surfaces and mucosal tissues (Ghafouri et al., 2024; Wu et al., 2025).

Additionally, certain postbiotic compounds particularly organic acids and structurally specific EPS derivatives may contribute to pH buffering and stabilization, counteracting rapid acidification caused by glycolytic activity of *S. mutans*. By mitigating the acidic microenvironment, postbiotics facilitate a shift toward a less cariogenic microbial phenotype and support the expansion of alkali-producing species. Taken together, these mechanisms suggest that postbiotic interventions can promote restoration of a stable, health-associated microbial ecosystem, offering a targeted and potentially safer alternative to broad-spectrum antimicrobial therapies.

## Directing microbial interactions through engineering approaches

Advances in synthetic biology and biomaterials engineering are transforming strategies for modulating microbial interactions within the oral cavity using postbiotics. Engineered biosystems enable the design of targeted antimicrobial molecules, including modified bacteriocins, precision peptides, and tailored metabolite derivatives that selectively neutralize *S. mutans* while preserving beneficial commensal species (Table 1). These engineered postbiotics may exhibit enhanced stability, prolonged half-life, and improved specificity toward defined virulence pathways such as EPS synthesis, acid production, and quorum-sensing signaling. By targeting discrete molecular mechanisms, such approaches aim to minimize collateral disruption of the broader oral microbiome.

**TABLE 1:** Postbiotics in dental caries management: Targets, mechanisms, and delivery approaches.

Postbiotic type	Mechanism of action	Target	Application	References
Short-chain fatty acids (SCFAs)	Inhibit acid production, modulate pH, antimicrobial activity	*S. mutans*, biofilm	Mouthwash, toothpaste, lozenges	Burzykowska et al. (2026), Luo et al. (2024)
Organic acids (lactic, phenyllactic)	Reduce bacterial growth, inhibit EPS synthesis	Cariogenic bacteria	Mouthwash, gels, slow-release films	Jiang X. et al. (2024)
Bacteriocins	Disrupt membrane integrity, inhibit biofilm formation	*S. mutans*, *Lactobacillus* spp.	Lozenges, toothpaste, paraprobiotic formulations	Banakar et al. (2024)
Exopolysaccharides (EPS)	Interfere with biofilm adhesion, modulate quorum sensing	Biofilm matrix, *S. mutans*	Nanoparticles, mucoadhesive films	Homayouni Rad et al. (2023), OmerOglou et al. (2022)
Cell-free supernatant metabolites	Antimicrobial and anti-adhesive effects	Cariogenic pathogens	Mouthwash, gels, toothpaste	Zhao et al. (2023), Mishra et al. (2025)
Heat-killed probiotics (paraprobiotics)	Immunomodulation (↑ sIgA, ↓ cytokines), biofilm disruption	Host immunity, *S. mutans*	Toothpaste, lozenges, oral gels	Heidari et al. (2025), Kumkum et al. (2026)

**FIGURE 3:**
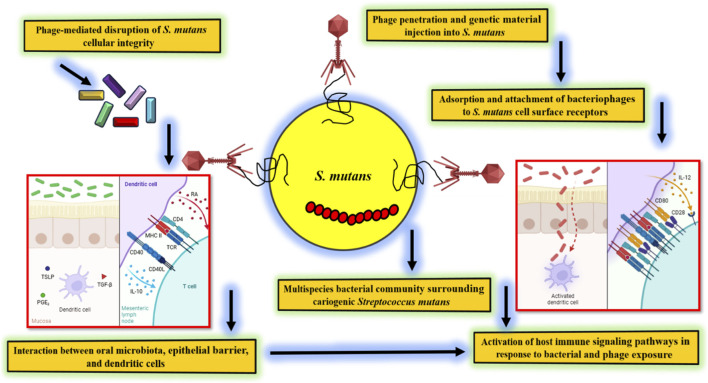
Bacteriophage host immune interactions targeting *S. mutans* in the oral environment.

Parallel developments in smart delivery platforms further strengthen the translational potential of postbiotic therapies. Nanocarrier-based formulations, mucoadhesive hydrogels, stimuli-responsive nanoparticles, and sustained-release lozenges can enable spatially and temporally controlled delivery of postbiotics within dental biofilms. Such systems enhance penetration through the EPS matrix, improve retention on tooth surfaces, and reduce salivary degradation, thereby increasing therapeutic efficacy (Figure 3). Emerging evidence also suggests that certain postbiotic metabolites may limit HGT among oral bacteria, potentially reducing dissemination of antimicrobial resistance and virulence determinants within *S. mutans* populations. Proposed mechanisms include interference with competence signaling pathways, inhibition of DNA uptake machinery, and disruption of phage-mediated gene exchange. Collectively, these engineering-based innovations illustrate how next-generation postbiotics can be harnessed to modulate oral microbial ecology with greater precision. By integrating synthetic design with advanced delivery technologies, such strategies offer a promising framework for preventing caries-associated dysbiosis while mitigating resistance evolution (Chen et al., 2025; Molina et al., 2025; Tiwari et al., 2024).

### Multi omics approaches

The integration of multi-omics technologies is transforming our understanding of how postbiotics remodel oral microbial communities and influence *S. mutans* mediated pathogenesis. Metagenomics enables comprehensive taxonomic profiling and functional characterization of the oral microbiome, allowing identification of shifts in commensal and pathogenic populations before and after postbiotic intervention. This approach facilitates mapping of microbial signatures associated with caries risk and prioritization of metabolic pathways that can be strategically targeted. Metatranscriptomics provides real-time insights into gene expression changes within microbial communities, offering deeper understanding of how postbiotics suppress virulence determinants in *S. mutans*, including acidogenicity, quorum sensing, EPS biosynthesis, and stress adaptation mechanisms.

Metabolomics complements these approaches by directly quantifying bioactive metabolites produced by postbiotics and resident commensals, thereby revealing metabolic rewiring, pH modulation, and inhibitory biochemical interactions at the molecular level. When integrated with network biology, these datasets enable identification of key regulatory hubs, microbial interaction nodes, and molecular switches that determine ecosystem stability or dysbiotic collapse. Such systems-level strategies not only enhance mechanistic insight but also support the rational design of precision postbiotic therapies tailored to individual microbiome profiles (Santana et al., 2024; Tong et al., 2025).

### Postbiotic phage interactions

The interaction between postbiotics and the oral virome represents an emerging Frontier in caries research. Growing evidence suggests that postbiotic metabolites can modulate bacteriophage activity and influence microbial community stability. Organic acids, antimicrobial peptides, and cell wall–derived fragments have been shown to alter phage infectivity and influence the balance between lytic and lysogenic cycles, thereby indirectly shaping *S. mutans* population dynamics.

Postbiotics may also modify bacterial surface receptors, enhancing or inhibiting phage adsorption and altering infection efficiency. Furthermore, postbiotic-induced changes in intracellular pH and redox conditions can influence prophage induction or lysogenic maintenance, ultimately affecting horizontal gene transfer dynamics within oral biofilms (Jiang S. et al., 2024). Notably, certain bacteriocins may increase phage susceptibility in *S. mutans* by disrupting membrane integrity and impairing stress-response pathways that typically confer resistance to phage infection. Collectively, these findings highlight the dual role of postbiotics as antimicrobial agents and modulators of phage bacteria interactions, contribution a different avenue to regulate dysbiosis and limit evolutionary adaptability of cariogenic pathogens (Sun et al., 2024).

### Combined phage and postbiotic therapeutic strategies

The combinatorial use of bacteriophages and postbiotics is emerging as a highly targeted strategy to control *S. mutans* and restore ecological balance within the oral microbiome. Postbiotic metabolites including organic acids, bacteriocins, and cell-free supernatants can sensitize *S. mutans* to phage infection by disrupting stress-response pathways, compromising membrane integrity, and weakening EPS-mediated biofilm protection. In addition to whole phages, phage-derived enzymes such as endolysins (which degrade peptidoglycan) and depolymerases (which break down EPS matrices) can synergize with postbiotics to penetrate mature cariogenic biofilms, enhance antimicrobial accessibility, and reduce community-level resistance. Because these enzymes do not rely on phage replication, they offer a controllable and potentially safer therapeutic alternative (Wei et al., 2024; Hasan and Aggarwal, 2025).

Advances in synthetic biology now permit engineering of phages to deliver antimicrobial biosynthetic genes, quorum-sensing inhibitors, antimicrobial peptides, or pH-modulating metabolites directly into the biofilm microenvironment. These engineered phages can function as programmable delivery vehicles, increasing local concentrations of postbiotic molecules while minimizing off-target effects on commensal communities. Such integrative phage postbiotic strategies represent a promising next-generation precision therapeutic framework capable of attenuating *S. mutans* virulence, restoring microbial equilibrium, and overcoming resistance associated with conventional antimicrobials (Sabarees et al., 2025; Ziegert et al., 2024).

### Viral bacterial co-dysbiosis perturbation

Postbiotics present significant potential for modulating viral bacterial co-dysbiosis, a complex ecological imbalance in which bacteriophages, pathogenic bacteria, and host-associated viruses collectively contribute to caries progression. One critical mechanism involves regulation of phage-encoded virulence gene transfer, particularly horizontal dissemination of genes associated with acid tolerance, EPS biosynthesis, and antimicrobial resistance (Table 2). Postbiotic metabolites may exert selective pressure on cariogenic phage populations, suppressing phages that enhance *S. mutans* competitiveness while favoring lytic phages capable of disrupting pathogenic biofilms. Such reshaping of the phage community can shift the microbial equilibrium toward a structure that promotes commensal stability rather than disease progression.

**TABLE 2:** Innovative phage postbiotic combination approaches: mechanisms, targets, and oral delivery systems for controlling cariogenic biofilms.

Therapeutic strategy	Mechanism	Target	Delivery	References
Postbiotic + lytic phages	Enhanced bacterial lysis, EPS disruption	*S. mutans* biofilm	Lozenges, gels, slow-release films	Ghamari et al. (2025), Zhu et al. (2025)
Phage-derived enzymes + postbiotics	Endolysins degrade cell wall; depolymerases dismantle EPS	Cariogenic biofilms	Nanoparticles, mouthwash	Rahman et al. (2025), Hwang et al. (2025)
Engineered phages delivering postbiotic genes	Local production of antimicrobial peptides, quorum-sensing inhibitors	*S. mutans*	Mucoadhesive films, oral gels	Qin et al. (2026), Isaac et al. (2026), Alparslan et al. (2026)

Beyond bacteriophage interactions, postbiotics may also influence the interplay between oral bacteria and host-associated viruses such as Herpes simplex virus (HSV) and Human papillomavirus (HPV), which have been implicated in modulating local inflammation and microbial behavior. Postbiotics can enhance epithelial barrier function, attenuate pro-inflammatory cytokine expression, and regulate mucosal immune responses, potentially reducing viral reactivation and the downstream alterations in bacterial virulence (Li et al., 2025; Guo X. et al., 2024; Yi et al., 2025). Collectively, these findings suggest that postbiotics may function as ecological modulators capable of simultaneously targeting bacterial virulence, phage dynamics, and host-viral interactions within the caries microenvironment.

### Dental caries prevention through postbiotic formulations

The incorporation of postbiotics into oral care formulations is gaining increasing attention as a preventive strategy for dental caries and oral microbiome stabilization. Conventional delivery systems including mouthwashes, toothpastes, lozenges, and gels are widely used for administering bioactive metabolites such as bacteriocins, organic acids, exopolysaccharides, and heat-inactivated probiotic derivatives. Mouthwashes enable rapid dispersion of postbiotics throughout the oral cavity, whereas toothpastes combine mechanical plaque removal with localized release of antimicrobial and anti-adhesive compounds. Mucoadhesive gels and lozenges provide prolonged retention within the oral cavity, increasing contact time with biofilms and enabling targeted suppression of *S. mutans* virulence traits (Iqbal et al., 2026; Nitya et al., 2026).

Advances in biomaterials engineering have facilitated the development of sustained-release delivery platforms, including nanoparticle carriers, polymeric films, and hydrogel matrices. These systems maintain therapeutic postbiotic concentrations within the biofilm microenvironment, enhance penetration into EPS-rich regions, and minimize rapid dilution by saliva. Sustained exposure supports prolonged antimicrobial activity and improved pH stabilization. Significantly, formulation design must ensure environmentally and dentally safe (ED-safe) concentrations. Postbiotics should be non-toxic to host tissues, preserve beneficial commensal populations, and retain chemical stability despite fluctuations in pH, salivary enzyme activity, and temperature. Together, these formulation strategies support the integration of postbiotics into routine oral care products and lay the groundwork for precision-based prevention of dental caries.

### Combined postbiotic and phage therapies

The integration of postbiotics with bacteriophage-based interventions represents a promising strategy to target resistant *S. mutans* biofilms and restore microbial balance within the oral cavity. Postbiotics including bacteriocins, organic acids, and CFS metabolites can weaken both the structural and physiological defenses of cariogenic biofilms, thereby enhancing phage penetration, adsorption, and lytic activity. This synergy addresses a key limitation of phage monotherapy, allowing phages to reach *S. mutans* cells embedded deep within the EPS matrix.

Conversely, phages can amplify the ecological impact of postbiotics by selectively lysing pathogenic strains, reducing pathogen colonization, and promoting the recolonization of commensal-dominated communities. The advent of multi-omics profiling and clinical microbiome analytics now enables the design of personalized phage postbiotic regimens tailored to an individual’s microbial composition, virulence gene expression patterns, and phage susceptibility profiles. Such precision approaches ensure optimal therapeutic dosing, minimize resistance development, and preserve beneficial microbial taxa. Overall, combined postbiotic and phage therapies offer a precision-based platform capable of disrupting robust biofilms, inhibiting pathogen repopulation, and addressing the heterogeneity of caries-associated dysbiosis across diverse patient populations.

### Dental plaque targeted delivery

Effective application of postbiotics against cariogenic biofilms requires precise delivery systems to maximize therapeutic efficacy while minimizing off-target effects on commensal microbiota. Nanoparticles enable targeted transport of postbiotic molecules, including bacteriocins, organic acids, and EPS, into the dense EPS matrix of *S. mutans* biofilms (Thayumanavan et al., 2025). These nanoscale carriers enhance penetration, protect labile metabolites from enzymatic degradation, and allow sustained release. Mucoadhesive films and hydrogels further improve retention on tooth surfaces and mucosal tissues, ensuring prolonged contact of postbiotics with the biofilm. Additionally, microencapsulation technologies can provide controlled release of postbiotics in response to environmental cues such as pH shifts, enzymatic activity, or microbial metabolites, selectively activating therapeutic action in cariogenic niches. Collectively, these delivery strategies provide localized, high-intensity exposure, destabilize biofilm structure, and support the growth of health-associated commensals, offering improved preventive and therapeutic management of dental caries.

### Host pathogen immunity map

The host–pathogen interface is essential for designing effective vaccines against *S. mutans*. The oral immune system features multiple defense layers, with salivary secretory immunoglobulin A (sIgA) serving as the first line of defense against bacterial adhesion, colonization, and toxins. sIgA plays a critical role in preventing early cariogenic microcolony formation. Beyond humoral immunity, pathogen-associated molecular patterns (PAMPs), including cell wall components and EPS of *S. mutans*, are recognized by TLRs on oral epithelial and immune cells. TLR signaling activates innate immune responses, cytokine production, and phagocyte recruitment, which collectively facilitate the transition to adaptive immunity. The oral virome also interacts with host immunity and bacterial communities, modulating inflammatory signaling, microbial gene expression, and bacterial resistance to immune clearance. Integrating these insights into a systems-level approach can guide rational vaccine design that not only induces robust mucosal and systemic immunity but also considers microbial–viral interactions to provide enhanced protection against *S. mutans*-associated caries.

## Postbiotics as vaccine adjuvants

Postbiotics are emerging as promising vaccine adjuvants, capable of modulating host immune responses without the risks associated with live microorganisms. Heat-killed bacterial cells, cell-wall fragments, EPS, and bacteriocin-derived peptides can enhance antigen presentation by stimulating dendritic cell maturation and activating cytokine signaling pathways. Specifically, EPS and bacteriocin fragments may function as immunostimulatory molecules, promoting mucosal immunity by increasing sIgA production and activating T cells at oral surfaces. By enhancing the recruitment of effector cells and amplifying both humoral and cellular responses to co-administered antigens, postbiotics can improve vaccine efficacy against *S. mutans*, potentially generating stronger immune protection in the absence of live microbial components (Table 3).

**TABLE 3:** Postbiotic-enhanced oral immunotherapeutic strategies targeting *S. mutans* virulence determinants.

Antigen/Target	Role in virulence	Postbiotic adjuvant	Delivery platform	References
SpaP (antigen I/II)	Adhesion to salivary pellicle	EPS fragments, heat-killed probiotics	Mucoadhesive nanoparticles, lozenges	Muhammad et al. (2026), Isaac et al. (2026)
GbpB	Glucan binding, biofilm formation	Bacteriocin fragments	Oral gels, films	Qin et al. (2026), Feng et al. (2026)
GtfB/GtfC	Glucosyltransferases, EPS synthesis	SCFAs, cell-free metabolites	Toothpaste, slow-release gels	Zhang Y. et al. (2025), Naz and Zafar (2025), Burzykowska et al. (2026)

## Future caries vaccine antigens

Rational design of caries vaccines requires identifying *S. mutans* antigens that are critical for adhesion, biofilm formation, and virulence. Key targets include SpaP (also known as P1 or I/II), GbpB, and PAc, which mediate bacterial attachment to the salivary pellicle and host tissues, thereby facilitating colonization. Enzymes involved in glucan synthesis, such as glucosyltransferases GtfB and GtfC, are also promising targets due to their central role in EPS production and biofilm structural integrity. Emerging research has highlighted bacteriocin-derived antigenic epitopes as a novel class of targets capable of eliciting immune responses against virulent strains while sparing commensal species. Collectively, these antigens offer multiple opportunities to develop vaccines that inhibit biofilm formation, prevent cariogenic colonization, and reduce the pathogenicity of *S. mutans*.

### Oral, nasal, and buccal delivery methods

Effective vaccination in the oral cavity requires delivery platforms that preserve antigen integrity and maximize mucosal exposure. Long-term retention on oral surfaces can be achieved using mucoadhesive nanoparticles and polymeric films, which enhance local antigen uptake and stimulate sIgA production. Heat-killed probiotic-based vectors are considered safe and can deliver antigens within an immunologically active environment to induce both innate and adaptive immune responses. Co-delivery of antigens with postbiotic adjuvants further enhances mucosal immunity by modulating cytokine responses, activating dendritic cells, and increasing protective sIgA production. Patient-friendly delivery methods, including oral rinses, lozenges, and nasal sprays, improve vaccine uptake, acceptability, and efficacy, offering practical strategies for preventive interventions against dental caries.

### Clinical evidence and translational considerations

#### Oral health clinical trials on postbiotics

Recent clinical studies have begun evaluating the effectiveness of postbiotics in oral health maintenance and dental caries prevention. Mouthrinses containing bacteriocins have been assessed for their ability to selectively reduce *S. mutans* populations, decrease plaque accumulation, and limit the growth of acidogenic biofilms, with several reports demonstrating significant reductions in cariogenic bacterial load without adversely affecting commensal species. Postbiotic formulations targeting EPS have also been investigated and shown to disrupt glucan-mediated adhesion and impair biofilm maturation *in vivo*. Furthermore, paraprobiotic toothpaste formulations containing heat-inactivated probiotic strains or cell-free supernatants have been reported to enhance oral microbial balance, increase salivary IgA levels, and reduce gingival inflammation. Collectively, these trials provide preliminary evidence that postbiotic interventions can be safely and effectively incorporated into preventive oral care programs.

#### Safety, stability, and regulatory considerations

The translation of postbiotic therapies into clinical practice requires careful evaluation of safety, production scalability, and regulatory compliance. Many postbiotic strains and their metabolites are classified as Generally Recognized as Safe (GRAS) for inclusion in consumer products. However, long-term safety must be considered, particularly regarding repeated exposure and potential impacts on the diversity of commensal oral microbiota. Formulation stability is critical to ensure consistent bioactivity, including resistance to enzymatic degradation, fluctuations in pH, and variations in temperature during storage and delivery. In addition, standardized production processes covering fermentation, metabolite extraction, and quality control are essential for commercial translation. Regulatory considerations, including accurate labeling, substantiation of efficacy claims, and adherence to national and international guidelines, must also be addressed to enable safe and effective implementation of postbiotic therapies in oral healthcare (Table 4).

**TABLE 4:** Advanced delivery systems for postbiotics in dental caries prevention.

Delivery system	Postbiotic cargo	Mechanism/ Advantage	Application
Nanoparticles	Bacteriocins, EPS, SCFAs	Biofilm penetration, sustained release	Mouthwash, gels
Mucoadhesive films	Heat-killed probiotics, bacteriocins	Prolonged retention on mucosa/tooth surface	Lozenges, oral films
Microencapsulation	Organic acids, SCFAs	Controlled release in response to pH or enzymes	Toothpaste, gels
Hydrogels	Bacteriocins, EPS inhibitors	Localized, slow release; protects postbiotics	Oral gels, slow-release inserts

## Limitations in virome research

The oral virome plays a significant role in microbial ecology and the development of dental caries. However, its therapeutic application is currently limited by several challenges. Detection of novel bacteriophages remains difficult due to the lack of comprehensive reference databases, the vast diversity of viral species, and the prevalence of lysogenic cycles, which are hard to identify using conventional sequencing approaches. Furthermore, the co-evolutionary dynamics between viruses and their bacterial hosts are poorly understood, particularly regarding the influence of phages on bacterial virulence, horizontal gene transfer, and the long-term stability of cariogenic biofilms. Addressing these gaps is critical to harnessing the oral virome in conjunction with postbiotic and phage-based therapies for precision oral health interventions.

### Future directions

#### Individualized oral microbiome engineering

Personalized microbiome engineering represents a promising Frontier in caries prevention and oral health management. Interventions can be tailored to an individual’s microbial composition and functional dynamics, enabling targeted modulation of the oral ecosystem. Rapid detection of dysbiotic signatures, virulence gene profiles, and phage susceptibilities can be achieved through high-throughput sequencing combined with AI-based diagnostic platforms. These data can guide the development of patient-specific postbiotic–phage combinations designed to selectively inhibit *S. mutans* and other cariogenic species while promoting beneficial commensals. Such precision approaches maximize therapeutic efficacy, minimize off-target effects, and reduce the likelihood of resistance or microbial rebound, moving beyond the traditional “one-size-fits-all” strategy in preventive dentistry.

#### Systems biology framework

Integration of multi-omics datasets including metagenomics, metatranscriptomics, metabolomics, and virome profiling within a systems biology framework allows comprehensive characterization of the oral microbial ecosystem and host–pathogen interactions. Modeling these complex networks alongside host immune parameters enables the identification of key regulatory nodes that control biofilm resilience and disease progression. Digital twin models are particularly promising, as they simulate patient-specific microbial, viral, and host interactions to forecast caries risk, predict treatment outcomes, and guide rational design of therapeutic interventions. Such computational platforms can accelerate the translation of laboratory findings into clinically actionable strategies.

### Next-generation postbiotics

#### Engineered metabolites targeting cariogenic pathways

Recent research has explored the development of bioactive metabolites designed to selectively interfere with major cariogenic determinants, including extracellular polysaccharide synthesis, acid production, and regulatory signalling networks. Targeting glucosyltransferase-mediated EPS formation or metabolic acidogenesis may allow attenuation of virulence traits while minimizing disruption to commensal microbial populations. However, most available evidence remains preclinical, and further validation in complex biofilm models and clinical settings is required.

### CRISPR-based paraprobiotic strategies

Advances in genome-editing technologies have enabled investigation into CRISPR-modified paraprobiotic systems, in which non-viable microbial cells are engineered to deliver defined antimicrobial peptides or gene-targeting components. These approaches are conceptually attractive because they combine functional specificity with reduced risk of uncontrolled microbial colonization. Nonetheless, challenges related to delivery efficiency, off-target effects, and regulatory approval must be addressed before clinical translation.

#### Quorum-sensing modulation

Postbiotic-derived quorum-sensing inhibitors represent another emerging strategy aimed at disrupting intercellular communication within *S. mutans* biofilms. By interfering with signalling pathways that regulate competence development, virulence gene expression, and biofilm maturation, these compounds may attenuate pathogenic behavior without exerting broad-spectrum antimicrobial pressure. Further mechanistic characterization and *in vivo* validation are needed to clarify their long-term ecological impact.

#### Translational considerations

Integration of engineered postbiotics with advanced delivery systems, bacteriophage-based interventions, and systems-level microbiome profiling may offer future opportunities for precision-based caries management. However, standardized efficacy testing, safety assessment, and regulatory frameworks remain essential prerequisites before such strategies can be implemented in clinical practice.

## Conclusion

Dental caries is a pervasive global health issue, arising from complex interactions among *S. mutans*, the oral microbiome, the virome, dietary habits, and host-related factors. Conventional preventive measures, although partially effective, are unable to selectively modulate microbial communities or address the ecological and evolutionary dynamics of cariogenic biofilms. Postbiotics have emerged as a promising class of targeted therapeutics, exhibiting antimicrobial activity, immunomodulatory effects, and the ability to reshape microbial and viral interactions without the risks associated with live probiotics. When combined with bacteriophage-based therapies, advanced delivery platforms, and multi-omics-guided interventions, postbiotics offer a robust strategy to disrupt resilient biofilms and restore oral microbial balance. Furthermore, the integration of systems biology and AI-directed diagnostics enables personalized approaches in oral care, where postbiotics, engineered molecules, and vaccine adjuvants can be employed to prevent and manage caries more effectively. Emerging clinical evidence and mechanistic insights underscore the potential of postbiotics as the foundation of next-generation oral health interventions, despite ongoing translational challenges. Future studies that integrate microbiome, virome, metabolome, and host immune analyses will be essential to fully realize the preventive and therapeutic potential of postbiotics, ultimately supporting safer, more practical, and sustainable strategies for global caries management.
